# The Incidence of Node-Positive Non-small-Cell Lung Cancer Undergoing Sublobar Resection and the Role of Radiation in Its Management

**DOI:** 10.3389/fonc.2020.00417

**Published:** 2020-05-26

**Authors:** John M. Varlotto, Isabel Emmerick, Rick Voland, Malcom M. DeCamp, John C. Flickinger, Debra J. Maddox, Christine Herbert, Molly Griffin, Paul Rava, Thomas J. Fitzgerald, Paulo Oliveira, Jennifer Baima, Rahul Sood, William Walsh, Lacey J. McIntosh, Feiran Lou, Mark Maxfield, Negar Rassaei, Karl Uy

**Affiliations:** ^1^Department of Radiation Oncology, University of Massachusetts Medical Center, Worcester, MA, United States; ^2^University of Massachusetts Medical School, Worcester, MA, United States; ^3^Division of Thoracic Surgery, University of Massachusetts Medical Center, Worcester, MA, United States; ^4^School of Nursing, University of Wisconsin, Madison, WI, United States; ^5^Division of Cardiothoracic Surgery, University of Wisconsin School of Medicine and Public Health, Madison, WI, United States; ^6^Department of Radiation Oncology, University of Pittsburgh Medical Center, Pittsburgh, PA, United States; ^7^Department of Medical Oncology, University of Massachusetts Medical Center, Worcester, MA, United States; ^8^Division of Pulmonary, Allergy and Critical Care Medicine, University of Massachusetts, Worcester, MA, United States; ^9^Department of Orthopedics and Rehabilitation, University of Massachusetts Medical Center, Worcester, MA, United States; ^10^Department of Radiology, University of Massachusetts, Worcester, MA, United States; ^11^Department of Pathology, Penn State Hershey Medical Center, Hershey, PA, United States

**Keywords:** sub-lobar resection (SLR), radiation, node positive, incidence, lung cancer

## Abstract

**Purpose:** To identify the incidence, preoperative risk factors, and prognosis associated with pathologically positive lymph node (pN+) in patients undergoing a sub-lobar resection (SLR).

**Methods:** This is a retrospective study using the National Cancer Database (NCDB) from 2004 to 2014 analyzing SLR excluding those with any preoperative chemotherapy and/or radiation, follow-up <3 months, stage IV disease, or >1 tumor nodule. Multivariable modeling (MVA) was used to determine factors associated with overall survival (OS). Propensity score matching (PSM) was used to determine preoperative risk factors for pN+ in patients having at least one node examined to assess radiation's effect on OS in those patients with pN+ and to determine whether SLR was associated with inferior OS as compared to lobectomy for each nodal stage.

**Results:** A total of 40,202 patients underwent SLR, but only 58.3% had one lymph node examined. Then, 2,615 individuals had pN**+** which decreased progressively from 15.1% in 2004 to 8.9% in 2014 (N1, from 6.3 to 3.0%, and N2, from 8.4 to 5.9%). A lower risk of pN+ was noted for squamous cell carcinomas, bronchioloalveolar adenocarcinoma (BAC), adenocarcinomas, and right upper lobe locations. In the pN+ group, OS was worse without chemotherapy or radiation. Radiation was associated with a strong trend for OS in the entire pN+ group (*p* = 0.0647) which was largely due to the effects on those having N2 disease (*p* = 0.009) or R1 resections (*p* = 0.03), but not N1 involvement (*p* = 0.87). PSM noted that SLR was associated with an inferior OS as compared to lobectomy by nodal stage in the overall patient population and even for those with tumors <2 cm.

**Conclusion:** pN+ incidence in SLRs has decreased over time. SLR was associated with inferior OS as compared to lobectomy by nodal stage. Radiation appears to improve the OS in patients undergoing SLR with pN+, especially in those with N2 nodal involvement and/or positive margins.

## Introduction

In 1995, a landmark investigation by the Lung Cancer Study Group demonstrated that sub-lobar resection (SLR) was inferior to lobectomy (tumor size <3 cm), reporting an increase in recurrence and trend toward decreased survival in patients undergoing SLR for non-small-cell lung cancer (NSCLC) (1). Subsequently, SLRs are often reserved for patients who cannot tolerate larger pulmonary resection (e.g., lobectomy) due to marginal pulmonary function tests and other comorbidities. Although there are several different definitions of adequate lymph node dissection (2), none pertains particularly to patients undergoing SLR.

Even with clinical stage I NSCLC in the era of PET-CT staging, past reports have indicated that as many as 10–20% of patients may have microscopic nodal involvement (3–6). Unfortunately, as many as 40–70% of patients undergoing SLR have not had a single lymph node removed or examined (7–10). Nevertheless, one recent retrospective review using the SEER database noted a lung cancer-specific survival (LCSS) and overall survival (OS) benefit with more extensive lymph node dissection in patients who received a sub-lobar excision (11).

The purpose of our study is to identify preoperative factors associated with lymph node involvement so that it could be better understood which patients would preferentially benefit from a lymphadenectomy and to assess whether patients with pathologically positive lymph nodes (pN+) may benefit from radiation therapy. We investigate the incidence of pN+ in patients undergoing SLR and whether SLR was associated with inferior OS in patients with pN+.

## Methods

Data for this study were derived from the National Cancer Database (NCDB) registry for patients diagnosed between 2004 and 2014. The NCDB currently captures 70% of all newly diagnosed malignancies in the United States annually. The patient information was de-identified. Thus, this investigation was exempted from institutional review board (IRB) approval. Individuals included were those undergoing SLR (sub-lobar NOS, wedge resection, segmentectomy) for stages I–III NSCLC. We also included patients undergoing (bi)lobectomy (*N* = 107,193) for the propensity score matching (PSM) to patients undergoing SLR so that the OS could be compared for each nodal level depending upon surgical resection. Exclusion criteria were any preoperative chemotherapy and/or radiation, follow-up <3 months, stage IV disease, >1 tumor nodule, or missing data on the timing of adjuvant therapy. To ensure that patients were receiving adjuvant and not salvage radiation, chemotherapy or postoperative radiation (PORT) had to be initiated within 120 days of surgery. PORT was permitted to start within 240 days after surgery if chemotherapy was started within the 120-day interval of surgery. Full selection criteria can be seen in Figure 4 in the Supplementary Material.

The outcomes were (a) pN+ involvement and (b) OS in those with lymph node involvement.

To identify the factors associated with pathologic lymph node involvement (among those who had any lymph node examined), it was performed as a bivariate and multivariate analysis considering the following variables: age, sex, race (non-Hispanic white, white Hispanic, black, other), location [right upper lobe (RUL), right middle lobe (RML), right lower lobe (RLL), left upper lobe (LUL), left lower lobe (LLL), other, NOS], histology [adenocarcinoma, bronchioloalveolar adenocarcinoma (BAC), adenosquamous cell carcinoma, large cell carcinoma, NSCLC NOS, squamous cell carcinoma], facility type (community program, academic research institute, comprehensive community cancer program, integrated), facility location (New England, East North Central, East South Central, Middle Atlantic, Mountain, Pacific, South Atlantic, West North Central, West South Central), insurance (unknown, private, Medicaid, Medicare, no insurance, government), income (< $38,000; $38,000–$48,000; >48,000–$63,000; >$63,000), area (rural near or not near metropolitan area, metropolitan area >1,000,000 population, metropolitan area > or <250,000, urban > or <20,000 near metropolitan area, urban > or <20,000 not near metropolitan area), type of resection (segmentectomy, wedge, sub-lobar NOS), Charlson comorbidity status as adapted by Deyo et al. (12) and tumor size.

To assess the effect of radiation on OS in those with pN+, all of the preoperative factors were considered. Additionally, the following factors were included: surgical margins (R0 clear, R1 microscopically positive, R2 grossly positive), lymphatic vascular invasion, tumor grade (well, moderate, poor, undifferentiated/anaplastic), number of nodes examined, number of pN+, radiation dose, chemotherapy, hospital readmission, length of stay, and T/N stage. These same factors for OS were also used in the PSM comparing lobectomy vs. SLR per N stage.

### Terminology

1. pN+ or node involvement or positive nodes refers to pathologic node involvement regardless of clinical enlargement, i.e., enlarged CT and/or fluorodeoxyglucose (FDG) avid.
2. cN+ refers to clinically enlarged nodes regardless of pathologic involvement. cN+ is the only term for clinical involvement. Of note, some patients could have had biopsy-positive nodes prior to surgical resection and be classified as clinical node involvement as per NCDB staging rules.

## Statistical Methods

The propensity match for the evaluation of factors predicting pN+ was performed only in the patients who had at least one node examined and clinically negative nodes. These propensity scores were created through logistic regression [area under the receiver operating characteristic (AUROC) = 0.8552] and matched for type of surgical resection and number of nodes examined.

The propensity match for comparison of the SLR and lobectomy groups by node stage were matched by age, sex, pathologic t-stage, number of nodes examined, number of positive nodes, tumor size, histology group (squamous, adenocarcinoma, and other), lymphovascular invasion (LVI), and Charlson comorbidity status.

Multivariate analysis for OS was performed in the pN+ population using proportional hazard Cox regression.

The propensity scores to assess the effects of radiation on the OS and the OS by surgical type by node stage were both performed only on patients with pN+ and matched by factors that were significant in the MVA for OS (age, sex, race, tumor location, histology, facility type, facility location, insurance status, income, urban/rural location, type of surgical resection, N stage, and tumor size). The Toolkit for Weighting and Analysis of Non-equivalent Groups (TWANG) macro was run in SAS to create the propensity scores for this pathologically node-positive group (13).

## Results

A total of 40,202 patients received SLR during the period analyzed. Then, 23,440 patients (58.3%) had at least one lymph node examined, and 2,615 of them were found to have pN+.

Of those having at least one node examined, the median number of nodes examined was 4 (1–83), and 11.2% of patients had positive nodes. Table 1 presents the percentage of patients with pN+ per analyzed variable for patients having at least one node examined. Facility type and location were significantly associated with having positive nodes. Academic research programs had a lower incidence of node involvement (10.28%) as compared to community hospitals and integrated network cancer programs (11.36–12.86%). Patients in New England had the lowest incidence of node involvement (9.14%), while the west south central area had the highest incidence of node involvement (15.08%). Personal characteristics (age, sex, and race) were all associated with node involvement. Patients who were younger than 65 years had a higher rate of nodal positivity than older (13.83 vs. 10.17%), while white non-Hispanic patients (10.82%) had the lowest incidence of all races, and women had a lower rate than men (10.48 vs. 12.01%). Both T stage and resection type (R0, R1, R2) were associated with a significant rise in node positivity with stage (T1, 8.93%; T2–T4, 18.45–18.27%), (R0, 10.09%; R1, 24.32%; R2, 34.11%). As differentiation decreased, so did the percentage of patients with nodal positivity (3.87–18.15%). Tumor size and lymphatic vascular invasion were also significantly associated with nodal positivity. Histology was significantly associated with nodal positivity with BAC (4.75%) and squamous cell carcinoma (8.53%) having the lowest risk, and large cell (16.16%) and NSCLC-NOS (18.44%) having the highest risk. Charles Mayo score was inversely associated with nodal positivity. Patients receiving their care through Medicare had the lowest rate of positive nodes (10.02%). SLR-NOS had a much greater rate of nodal positivity (19.1%) than segmentectomy (10.62%) and wedge resection (11.02%). There was no significant association with nodal positivity based upon geographic location or income.

**Table 1:** Percentage of patients having pathological nodal positivity by analyzed factor for patients having at least one node examined.

**Variable analyzed**		**Total patient numbers**	**Patients without positive nodes (%)**	**Patients with positive nodes (%)**	***P***
Facility type	Academic/Research program	10,235	9,183 (89.7%)	1,052 (10.3%)	0.0014
	Community cancer program	1,376	1,199 (87.1%)	177 (12.9%)	
	Comprehensive community cancer program	9,636	8,503 (88.2%)	1,133 (11.8%)	
	Integrated network cancer program	2,148	1,904 (88.6%)	244 (11.4%)	
Facility location	East North Central	4,354	3,896 (89.5%)	458 (10.5%)	< 0.0001
	East South Central	1,767	1,543 (87.3%)	224 (12.7%)	
	Middle Atlantic	4,962	4,454 (89.8)	508 (10.2%)	
	Mountain	743	659 (88.7%)	84 (11.3%)	
	New England	1,784	1,621 (90.9%)	163 (9.1%)	
	Pacific	2,046	1,835 (89.7%)	211 (10.3%)	
	South Atlantic	5,104	4,497 (88.1%)	607 (11.9%)	
	West North Central	1,594	1,400 (87.8%)	194 (12.2%)	
	West South Central	1,041	884 (84.9%)	157 (15.1%)	
Year of diagnosis	2004	747	634 (84.9%)	113 (15.1%)	< 0.0001
	2005	887	766 (86.4%)	121 (13.6%)	
	2006	986	838 (85.0%)	148 (15.0%)	
	2007	1,231	1,082 (87.9%)	149 (12.1%)	
	2008	2,070	1,814 (87.6%)	256 (12.4%)	
	2009	2,410	2,094 (86.9%)	316 (13.1%)	
	2010	2,647	2,343 (88.5%)	304 (11.5%)	
	2011	2,820	2,532 (89.8%)	288 (10.2%)	
	2012	3,002	2,686 (89.5%)	316 (10.5%)	
	2013	3,229	2,928 (90.7%)	301 (9.3%)	
	2014	3,411	3,108 (91.1%)	303 (8.9%)	
Race	Asian or Pacific Islander	359	311 (86.6%)	48 (13.4%)	0.0002
	Black	1,706	1,483 (86.9%)	223 (13.1%)	
	Other/Unknown	1,500	1,330 (88.7%)	170 (11.3%)	
	White Hispanic	415	347 (83.6%)	68 (16.4%)	
	White Non-hispanic	19460	17354 (89.2%)	2106 (10.8%)	
Primary payor	Insurance status unknown	267	223 (83.5%)	44 (16.5%)	< 0.0001
	Medicaid	905	781 (86.3%)	124 (13.7%)	
	Medicare	16,104	14,491 (90.0%)	1,613 (10.0%)	
	Not insured	293	252 (86.0%)	41 (14.0%)	
	Other Government	167	142 (85.0%)	25 (15.0%)	
	Private insurance	5,704	4,936 (86.5%)	768 (13.5%)	
Urban/Rural 2003	Completely rural or <2,500 urban population, adjacent to a metro area	239	214 (89.5%)	25 (10.5%)	0.1172
	Completely rural or <2,500 urban population, not adjacent to a metro area	245	217 (88.6%)	28 (11.4%)	
	Counties in metro areas of 1 million population or more	12,371	11,029 (89.2%)	1,342 (10.8%)	
	Counties in metro areas of 250,000–1 million population	4,246	3,727 (87.8%)	519 (12.2%)	
	Counties in metro areas of fewer than 250,000 population	2,135	1,903 (89.1%)	232 (10.9%)	
	Urban population of 2,500–19,999, adjacent to a metro area	1,268	1,106 (87.2%)	162 (12.8%)	
	Urban population of 2,500–19,999, not adjacent to a metro area	637	575 (90.3%)	62 (9.7%)	
	Urban population of 20,000 or more adjacent to a metro area	1,151	1,030 (89.5%)	121 (10.5%)	
	Urban population of 20,000 or more not adjacent to a metro area	341	296 (86.8%)	45 (13.2%)	
Median income quartiles 2000	$30,000–$35,999$36,000–$45,999$46,000+ < $30,000	3,9296,1039,9302,661	3,479 (88.6%)5,407 (88.6%)8,870 (89.3%)2,333 (87.7%)	450 (11.4%) 696 (11.4%)1,060 (10.7%)328 (12.3%)	0.0837
Charlson-deyo score	012	10,5898,8953,956	9,310 (87.9%)7,927 (89.1%)3,588 (90.7%)	1,279 (12.1%)968 (10.9%)368 (9.3%)	< 0.0001
Surgery type	Segmentectomy	5,508	4,923 (89.4%)	585 (10.6%)	< 0.0001
	Sub-Lobar resection, NOS	665	538 (80.9%)	127 (19.1%)	
	Wedge resection	17,267	15,364 (89.0%)	1,903 (11.0%)	
Tumor location	Left lower lobe	3,508	3,157 (90.0%)	351 (10.0%)	< 0.0001
	Left upper lobe	7,064	6,143 (87.0%)	921 (13.0%)	
	Main bronchus	28	24 (85.7%)	4 (14.3%)	
	Other/NOS	638	534 (83.7%)	104 (16.3%)	
	Right lower lobe	4,272	3,795 (88.8%)	477 (11.2%)	
	Right middle lobe	706	612 (86.7%)	94 (13.3%)	
	Right upper lobe	7,224	6,560 (90.8%)	664 (9.2%)	
Histology	Adenocarcinoma	13,474	11,805 (87.6%)	1,669 (12.4%)	< 0.0001
	Adenosquamous	621	531 (85.5%)	90 (14.5%)	
	BAC	1,684	1,604 (95.2%)	80 (4.8%)	
	Large cell CA	724	607 (83.8%)	117 (16.2%)	
	Non-small-cell carcinoma, NOS	678	553 (81.6%)	125 (18.4%)	
	Squamous cell CA	6,259	5,725 (91.5%)	534 (8.5%)	
T-Stage	T1	17,971	16,366 (91.1%)	1,605 (8.9%)	< 0.0001
	T2	4,043	3,297 (81.6%)	746 (18.4%)	
	T3	720	585 (81.2%)	135 (18.8%)	
	T4	706	577 (81.7%)	129 (18.3%)	
Surgical Margins	R0	22,043	19,819 (89.9%)	2,224 (10.1%)	< 0.0001
	R1	699	529 (75.7%)	170 (24.3%)	
	R1 or R2	569	392 (68.9%)	177 (31.1%)	
	R2	129	85 (65.9%)	44 (34.1%)	
Gender	Female	13,082	11,711 (89.5%)	1,371 (10.5%)	0.0002
	Male	10,358	9,114 (88.0%)	1,244 (12.0%)	
Lymph vascular invasion	Not presentPresentUnknown	11,6002,1731,336	10,898 (94.0%)1,564 (72.0%)1,135 (85.0%)	702 (6.0%)609 (28.0%)201 (15.0%)	< 0.0001
Grade	Cell type not determined, not stated or not applicable, unknown primaries, high grade dysplasia	1,500	1,307 (87.1%)	193 (12.9%)	< 0.0001
	Well-differentiated	3,796	3,649 (96.1%)	147 (3.9%)	
	Moderately differentiated, moderately well-differentiated, intermediate differentiation	10,576	9,510 (89.9%)	1,066 (10.0%)	
	Poorly differentiated	7,287	6,129 (84.1%)	1,158 (15.9%)	
	Undifferentiated, anaplastic	281	230 (81.8%)	51 (18.2%)	
Age	<65	6,314	5,441 (86.2%)	873 (13.8%)	< 0.0001
	≥65	17,126	15,384 (89.8%)	1,742 (10.2%)	
Tumor size	<20 mm	12,168	11,245 (92.4%)	923 (7.6%)	< 0.0001
	≥20 mm	11,272	9,580 (85.0%)	1,692 (15.0%)	

When clinically node-negative patients with (5.2% of these patients) and without pathologic node involvement were matched by PSM for type of resection and number of nodes examined, the factors that were associated with pN**+** are listed by the factor and its associated odds ratio. Non-RUL locations remained significantly associated with node involvement: LLL, 1.59 (1.28–1.97); LUL, 1.71 (1.42–2.06); RLL, 1.77 (1.44–2.17); RML, 1.78 (1.20–2.64); and other locations, 2.50 (1.70–3.70). Likewise, squamous cell carcinoma 0.51 (0.43–0.62) and BAC 0.34 (0.23–0.49) histologies were significantly less likely to have node involvement, and tumor size was positively associated with node involvement 1.02 (1.014–1.031). However, non-traditional factors associated with nodal involvement included comprehensive community cancer center vs. academic research program, 1.18 (1.012–1.374), and white Hispanic vs. white non-Hispanic ethnicity, 1.68 (1.11–2.54). A total of 762 patients had clinical N1, 930 had clinical N2, and 82 had clinical N3 involvement, and the percentage of patients with pathologic lymph node involvement was 64, 71, and 38%, respectively.

**Figure 1:**
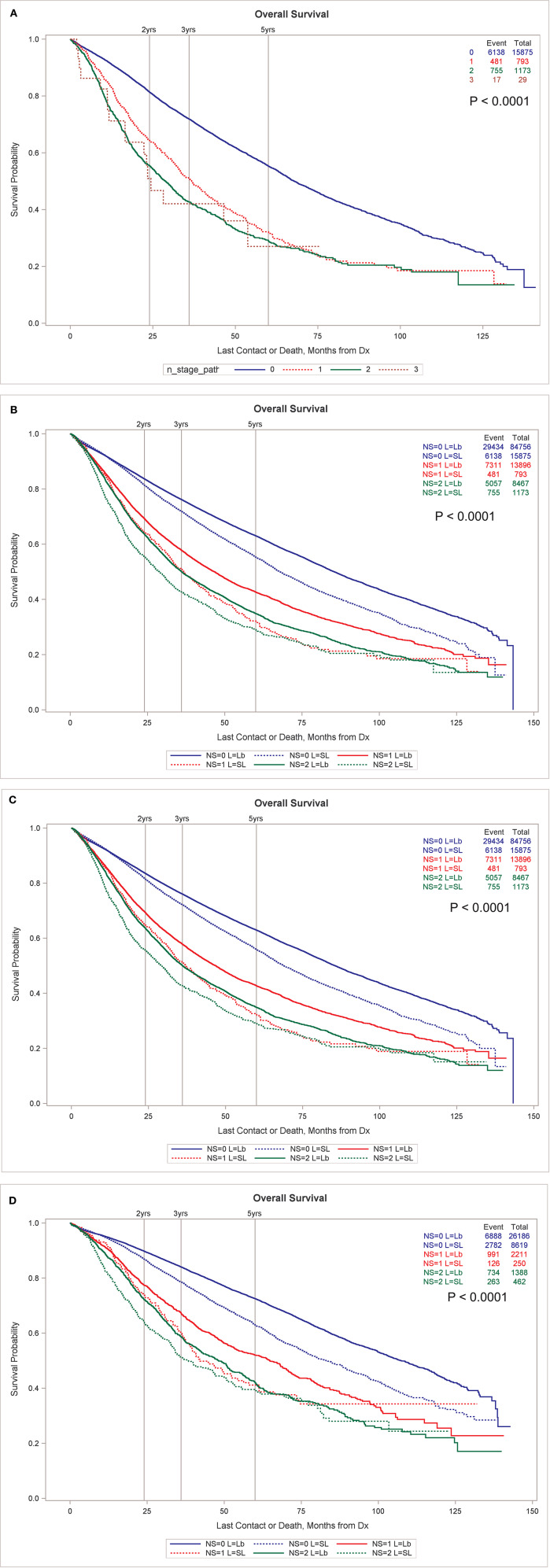
**(A)** OS by node stage in patients undergoing SLR. **(B)** OS by node stage by type of resection, sub-lobar vs. lobectomy, unmatched. Propensity match for OS by node stage by type of resection for all resected tumors **(C)** and only for those <2 cm in size **(D)**. All node stages are pathologic in this figure. **(B–D)** exclude N3 nodes for clarity. **(B–D)** Including N3 nodes are available in Supplementary Material.

The OS of patients receiving SLR and at least one node examined by node stage can be seen in Figure 1A. There are 15,875, 793, 1,173, and 29 patients with N0, N1, N2, and N3 nodes. Despite the inadequate surgical treatment of the primary tumor, the OS still sharply decreased by node stage. Figure 1B shows that OS by node stage is significantly less for the SLR group compared to the lobectomy group without propensity matching. After propensity matching, the SLR group still has lower OS for the entire surgical population (Figure 1C) and for the subset of patients with tumors < = 2 cm (Figure 1D). See Supplementary Material for Figures S1B–D including N3 nodes.

Table 2 demonstrates that the risk of patients having pN+ decreased during the years of our study from 2004 to 2014 in all node stages. The risk of any positive nodes (15.13–8.88%) as well as N1 nodes (6.29–2.96%), N2 (8.43–5.86%), and N3 (0.4–0.06%) all decreased during the years of our study. Supplemental Table 2 notes the incidence of pN+ in patients who are not cN+.

**Table 2:** Percentage of patients with pathologically N1, N2, and N3 node involvement during years 2004–2014.

**Year of diagnosis**	**Total patients/Year**	**N0 Freq (%)**	**Total positive nodes per year (%)**	**N1 Freq (%)**	**N2 Freq (%)**	**N3 Freq (%)**
2004	747	634 (84.9)	113 (15.1)	47 (6.3)	63 (8.4)	3 (0.4)
2005	887	766 (86.4)	121 (13.6)	47 (5.3)	69 (7.8)	5 (0.6)
2006	986	838 (85.0)	148 (15.0)	71 (7.2)	76 (7.7)	1 (0.1)
2007	1,231	1,082 (87.9)	149 (12.1)	65 (5.3)	83 (6.7)	1 (0.1)
2008	2,070	1,814 (87.6)	256 (12.4)	108 (5.2)	142 (6.9)	6 (0.3)
2009	2,410	2,094 (86.9)	316 (13.1)	100 (4.2)	211 (8.8)	5 (0.2)
2010	2,647	2,343 (88.5)	304 (11.5)	117 (4.4)	180 (6.8)	7 (0.3)
2011	2,820	2,532 (89.8)	288 (10.2)	112 (4.0)	174 (6.2)	2 (0.1)
2012	3,002	2,686 (89.5)	316 (10.5)	122 (4.1)	190 (6.3)	4 (0.1)
2013	3,229	2,928 (90.7)	301 (9.3)	106 (3.3)	191 (5.9)	4 (0.1)
2014	3,411	3,108 (91.1)	303 (8.9)	101 (3.0)	200 (5.9)	2 (0.1)

Multivariate analysis for OS for patients having pN**+** can be seen in Table 3. Supplemental Table 3 includes the MVA for OS in patients having only pN+ without cN+. Personal characteristics associated positively with OS include younger age, female sex, Asian/Pacific Islander vs. non-Hispanic white, and having a 0 for Charlson comorbidity status. Histologic factors associated with survival included tumor size, number of positive nodes, number of nodes examined, higher T stage, poor differentiation, and lymphatic vascular invasion. Treatment factors associated with OS include not having radiation or chemotherapy and length of stay.

**Table 3:** Multivariate analysis for overall survival (OS) in the pathologic node-positive group.

**Description**	**Hazard ratio**	**Lower 95% Wald**	**Upper 95% Wald**	***P*-value**
		**Confidence limit**	**Confidence limit**	
**AGE (/10 years)**	**1.290**	**1.153**	**1.442**	< 0.0001
**SEX Male vs. Female**	**1.231**	**1.086**	**1.396**	0.001
**race: Asian or Pacific Islander vs. White Non-hispanic**	**0.492**	**0.283**	**0.855**	0.02
race_group5 Other/Unknown vs. White Non-hispanic	0.815	0.639	1.04	
race: Other/Unknown vs. White Non-hispanic	0.957	0.761	1.204	
race: White Hispanic vs. White Non-hispanic	0.647	0.405	1.036	
**CDCC: 1 vs. 0**	**1.174**	**1.027**	**1.343**	0.03
**CDCC: 2 vs. 0**	**1.209**	**1.003**	**1.459**	
Location: Left lower lobe vs. Right upper lobe	0.975	0.794	1.197	0.84
Location: Left upper lobe vs. Right upper lobe	0.983	0.836	1.155	
Location Main bronchus vs. Right upper lobe	Undef	0	7.23E + 103	
Location: Other/NOS vs. Right upper lobe	1.178	0.842	1.649	
Location: Right lower lobe vs. Right upper lobe	1.062	0.878	1.284	
Location: Right middle lobe vs. Right upper lobe	1.179	0.842	1.65	
histology: Adenosquamous vs. Adenocarcinoma	1.15	0.844	1.567	0.85
histology: BAC vs. Adenocarcinoma	1.136	0.811	1.591	
histology: Large cell CA vs. Adenocarcinoma	1.029	0.724	1.463	
histology: Non-small-cell carcinoma vs. Adenocarcinoma	1.144	0.868	1.507	
histology: Squamous cell CA vs. Adenocarcinoma	1.042	0.889	1.222	
Facility_Type: Community cancer program vs. academic/research program	0.992	0.754	1.305	0.56
Facility_Type: Comprehensive community cancer program vs. Academic/Research program	1.026	0.891	1.182	
Facility_Type: Integrated network cancer program vs. Academic/research program	0.871	0.694	1.092	
Facility_Location: East North Central vs. New England	0.996	0.741	1.338	0.47
Facility_Location: East South Central vs. New England	0.912	0.655	1.269	
Facility_Location: Middle Atlantic vs. New England	0.9	0.672	1.207	
Facility_Location: Mountain vs. New England	0.647	0.409	1.023	
Facility_Location: Pacific vs. New England	1.077	0.77	1.506	
Facility_Location: South Atlantic vs. New England	1.003	0.752	1.339	
Facility_Location: West North Central vs. New England	0.952	0.679	1.335	
Facility_Location: West South Central vs. New England	1.061	0.743	1.515	
Insurance Status: Unknown vs. Private insurance	1.08	0.571	2.044	0.43
Insurance Status: Medicaid vs. Private insurance	1.134	0.825	1.56	
Insurance Status: Medicare vs. Private insurance	0.871	0.732	1.037	
Insurance Status: Not Insured vs. Private insurance	0.936	0.564	1.552	
INSURANCE_ Other Government vs. Private insurance	1.295	0.694	2.416	
Median Income 2012: $38,000–$47,999 vs. < $38,000	0.909	0.749	1.104	0.086
Median Income 2012: $48,000–$62,999 vs. < $38,000	0.9	0.736	1.101	
MED_INC_QUAR_ $63,000 + vs. < $38,000	0.767	0.619	0.951	
Completely rural or <2,500 urban population, adjacent to a metro area vs. Counties in metro areas of 1 million population or more	0.639	0.333	1.229	0.36
Completely rural or <2,500 urban population, not adjacent to a metro area vs. Counties in metro areas of 1 million population or more	1.149	0.627	2.104	
Counties in metro areas of 250,000–1 million population vs. Counties in metro areas of 1 million population or more	1.154	0.981	1.357	
Counties in metro areas of fewer than 250,000 population vs. Counties in metro areas of 1 million population or more	1.103	0.88	1.382	
Urban population of 2,500–19,999, adjacent to a metro area vs. Counties in metro areas of 1 million population or more	1.19	0.92	1.54	
Urban population of 2,500–19,999, not adjacent to a metro area vs. Counties in metro areas of 1 million population or more	0.867	0.566	1.328	
Urban population of 20,000 or more adjacent to a metro area vs. Counties in metro areas of 1 million population or more	1.074	0.793	1.453	
Urban population of 20,000 or more not adjacent to a metro area vs. Counties in metro areas of 1 million population or more	0.851	0.536	1.351	
Surgery: Segmentectomy vs. Wedge resection	0.953	0.822	1.106	0.78
Surgery: Sub-lobar resection, NOS vs. Wedge resection	0.941	0.7	1.264	
**Tumor Size (/10 cm)**	**1.057**	**1.028**	**1.087**	0.0001
**Any Pathologically Positive Nodes vs. None**	**1.102**	**1.063**	**1.143**	< 0.0001
**Nodes examined by pathology**	**0.968**	**0.957**	**0.979**	< 0.0001
Year of diagnosis	0.966	0.919	1.016	0.18
**Pathological T-Stage: T2 vs. T1**	**1.208**	**1.054**	**1.385**	0.009
Pathological T-Stage: T3 vs. T1	1.107	0.869	1.41	
**Pathological T-Stage: T4 vs. T1**	**1.46**	**1.092**	**1.953**	
**N2 vs. N1**	**1.291**	**1.125**	**1.482**	0.0003
Radiation: Any vs. None	1.345	0.905	2	0.14
**RAD_REGIONAL_DOSE_cGy Unit** **=** **10,000**	**0.411**	**0.194**	**0.873**	0.021
**chemo_ Any vs. None**	**0.777**	**0.667**	**0.906**	0.001
**LVI _ Present vs. Not present**	**1.446**	**1.188**	**1.759**	0.001
LVI_ Unknown vs. Not present	1.172	0.945	1.454	
GRADE: Cell type not determined, not stated or not applicable, unknown primaries, high-grade dysplasia vs. well-differentiated, differentiated, NOS	0.928	0.623	1.382	0.010
GRADE: Moderately differentiated, moderately well-differentiated, intermediate differentiation vs. well-differentiated, differentiated, NOS	1.234	0.922	1.652	
**GRADE: Poorly differentiated vs. well-differentiated, differentiated, NOS**	**1.394**	**1.04**	**1.869**	
GRADE: Undifferentiated, anaplastic vs. well-differentiated, differentiated, NOS	1.664	0.949	2.919	
Hospital readmission: Planned and unplanned readmission within 30 days of discharge vs. patient not readmitted	1.741	0.543	5.585	0.31
Hospital readmission: Planned readmission within 30 days of discharge vs. patient not readmitted	1.264	0.908	1.76	
Hospital readmission: Unknown if surgery recommended/performed, unknown if readmitted within 30 days of discharge vs. patient not readmitted	0.84	0.541	1.303	
Hospital readmission: Unplanned readmission within 30 days of discharge vs. patient not readmitted	1.188	0.899	1.571	
**Surgical Discharge (/day)**	**1.026**	**1.018**	**1.034**	< 0.0001

**Figure 2:**
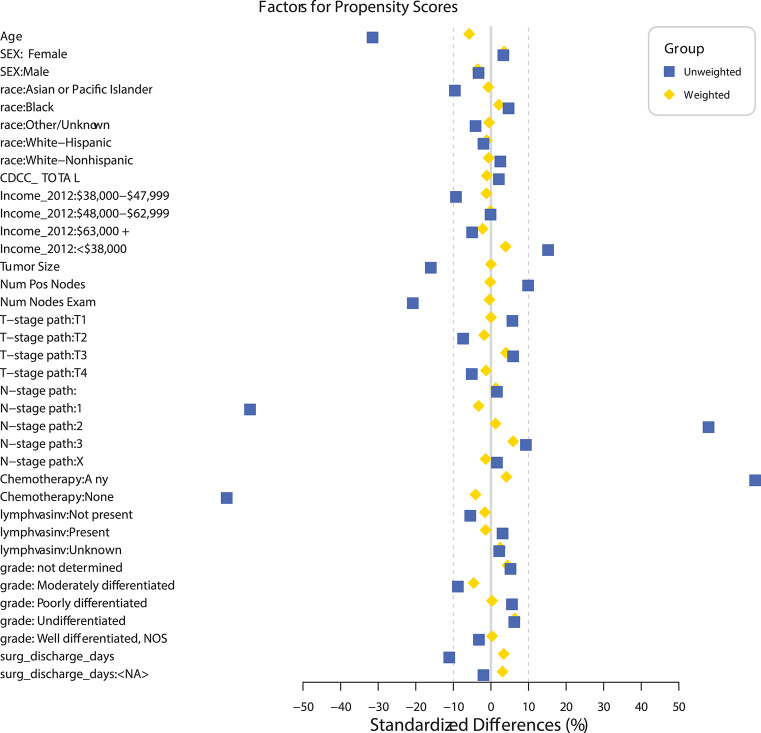
Standardized differences before and after propensity matching between the groups receiving and not receiving radiation in patients undergoing sub-lobar resection with at least one pathologically positive lymph node (pN+).

**Figure 3:**
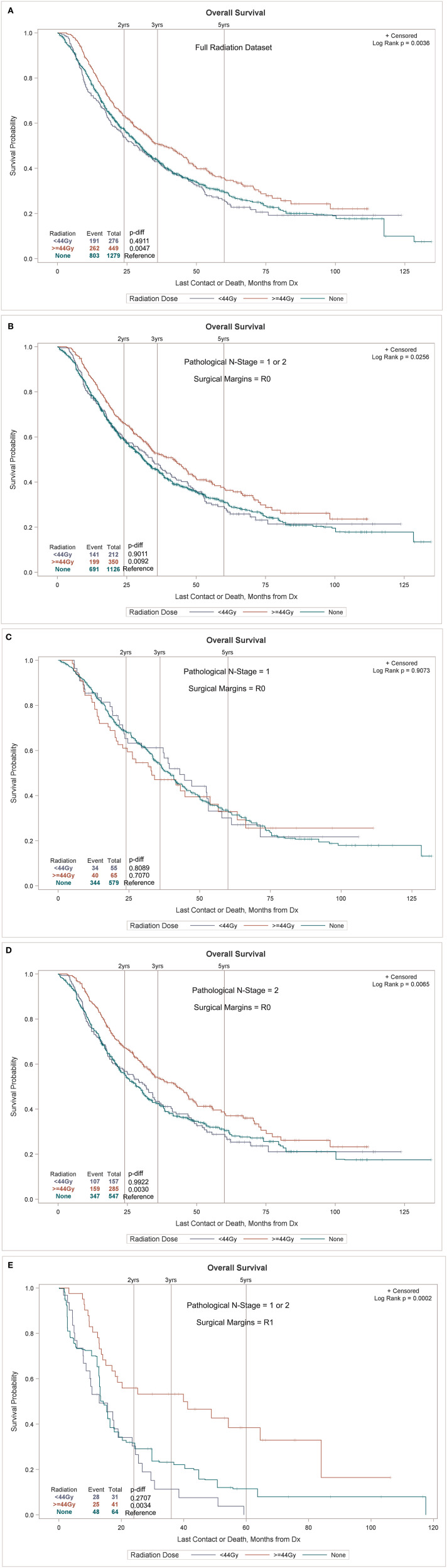
Overall survival (OS) of the sub-lobar resection (SLR) group with pathologically positive lymph node (pN+) with or without radiation in the entire node-positive population **(A)** and in the subgroups with involvement of both N1 and N2 node involvement **(B)**, N1 node involvement **(C)**, N2 node involvement **(D)**, and with positive surgical margins **(E)**.

A propensity match to assess the effects of radiation on OS was performed only on patients with pN+ and matched by factors that were significant in the MVA for OS (age, sex, race, tumor location, histology, facility type, facility location, insurance status, income, urban/rural location, type of surgical resection, N stage, and tumor size). The standardized differences between the patients receiving and not receiving radiation before and after the match can be seen in Figure 2, demonstrating the successful alignment of prognostic factors after the match. After propensity matching, OS curves were generated for the entire population and the subgroups with negative margins and positive margins, as can be seen in Figures 3A–E. When an adequate radiation dose of >44 Gy was given, OS was significantly better in the entire node-positive group (*p* = 0.0047; Figure 3A), those with either N1 or N2 nodal involvement and R0 resection (0.0092; Figure 3B), those with N2 nodal involvement and R0 resection (*p* = 0.0030; Figure 3D), and those with N1 or N2 node involvement and an R1 resection (*p* = 0.0034; Figure 3E), but not in those with N1 nodal involvement and R0 resection (*p* = 0.707; Figure 3C).

## Discussion

During the years of our study 2004–2014, 40,202 patients underwent SLR, but similar to another recent series of patients receiving SLR, only a small majority of patients had at least one lymph node examined, 58.3% in our population and 55.4% in the past series (11). Then, 2,615 patients or 11.2% of the 23,440 patients having one node examined had at least one pN+. The percentage of patients with pN+ decreased steadily during the years of our study from 15.1 to 8.9%. Likewise, the percentage of pN+ decreased in patients with both N1 and N2 disease.

The propensity match for preoperative factors for pN+ did not include differentiation because this information is often not present with biopsies and can be inaccurate. Small biopsies often contain scant tumor cells, crush artifact, and/or necrosis, so differentiation is often difficult to obtain (14). We did not include cN+ in our models because we feel that all clinically enlarged nodes should be assessed for nodal involvement prior to consideration of any planned surgical procedure. Our investigation noted that of the patients presenting with clinically enlarged nodes, 66.5% had positive nodes. Central location is a known risk factor for lymph node involvement, but this risk factor was not readily available for all populations in this large database, and it should be noted that this definition does vary in the literature (15). Furthermore, one recent large series of 938 patients noted that those with centrally located tumors (defined the inner two thirds of the lung on CT) were not at increased risk of lymph node involvement (16). Our propensity match noted that white Hispanic patients have an increased risk of node involvement. There is conflicting literature in regard to white Hispanic patients' risk of node involvement compared to other races (17, 18). We do not know of any other series that demonstrates that the RUL location has a lower risk of node involvement except for one series using the SEER database that demonstrated a significantly lower risk of node involvement with right upper as compared to right lower lobe locations (18). Since central location is associated with a higher chance of lymph node involvement (19) and this factor is unavailable in NCDB, we speculate that the RUL location possibly had more tumors located peripherally within the lung. Of course, smaller tumor size (20) and BAC have been associated with a lower risk of lymph node involvement (21). However, it must be acknowledged that BAC as classified in this database is a heterogeneous category since a multi-society committee reclassifed BAC into new categories of adenocarcinoma *in situ* (AIS), minimally invasive adenocarcinoma (MIA), invasive lepidic adenocarcinoma, and invasive mucinous adenocarcinoma (22), so we expect tumors classified as BAC in our project to represent a heterogeneous group with a relatively low risk of pN+. Squamous cell carcinoma was also found to have a lower risk of node involvement as noted previously (23–25). Comprehensive community cancer centers had a higher risk of lymph node involvement [odds ratio (OR) = 1.18] as compared to those undergoing SLRs at an academic research institution.

In our population of patients with SLR having at least one node examined, it was noted that the nodal stage was still predictive of OS, with OS decreasing as pathologic nodal stage increased. Even after propensity matching the patients with SLR to those with lobectomy, it was noted that OS with SLR was significantly less for all pathologic node stages. This indicates that suboptimal surgery is a major prognostic factor that is associated with a survival decrement regardless of the extent of node involvement.

Our PSM for radiation was well-balanced for patients receiving and not receiving radiation and was matched for all major prognostic factors including LVI, age, gender, chemotherapy use, tumor size, T stage, N stage, Charlson comorbidity status, tumor grade, number of positive nodes, etc. When analyzed by an adequate radiation dose of >44 Gy, radiation was associated with a significant survival increase in all patients with pathologically involved nodes, particularly those with positive margins and N2 involvement. Radiation had no effect on OS in the patients with margin-negative disease with pathologic N1 involvement.

The role of postoperative radiation has not been definitely proven in patients with or without optimal surgical resection. We know of no other studies assessing the role of radiation therapy in patients treated with SLR with pN+. There are currently no recommendations for this situation. Because of the limited number of patients with SLR who are found to have positive nodes, the role of radiation in this setting will likely never be proven. Our investigation has noted that similar to patients undergoing lobectomy/pneumonectomy, OS decreases with higher pathologic nodal stage. Because our results echo the current National Comprehensive Cancer Network (NCCN) guideline recommendations concerning PORT in patients receiving optimal surgery, we believe SLR does not change the current indications for PORT despite the known inferior OS and local recurrence risk in this situation. The NCCN guidelines note that PORT appears to improve OS in patients with N2 disease and/or positive margins but states that radiation is not recommended for N0 or N1 disease (26). The PORT meta-analysis (27) of prospective trials using outdated radiation techniques demonstrated a possible survival benefit noted in the N2 population (26) in the era prior to the proven role of adjuvant chemotherapy. Similarly, a retrospective study-assembled data prior to the proven role of adjuvant chemotherapy also noted the beneficial effects of radiation on survival in those having N2 nodal involvement (28). However, in the setting of adjuvant chemotherapy and better staging, the beneficial role of radiation in patients having N2 involvement have been mixed in more recent retrospective studies (29–31). We speculate that these more modern studies did not assess patients by factors associated with local recurrence which caused the variable results. Indeed, one recent retrospective series noted that node stage was related to distal recurrence and OS, but not local recurrence (32). In regard to positive margins, radiation therapy has been suggested to improve OS whether it is given concurrently or after chemotherapy (33, 34). Of interest, a recent randomized phase II study assessed the benefits of postoperative concurrent chemo/radiation vs. post-operative chemotherapy only in patients undergoing complete resection [>90% (bi)lobectomy] of N2 involved NSCLC and noted no OS or disease-free survival benefit to radiation therapy (35). Due to small patient numbers in our study, the question of sequencing could not be entertained in our investigation.

There are many shortcomings with our investigation. The data used in this study are retrospective, but they cover ~70% of all newly diagnosed malignancies in the United States annually. Important information is missing such as the rationale for treating these patients with SLR and the presurgical performance status. Furthermore, it is not known why some patients received suboptimal doses of radiation. Suboptimal radiation may have been given due to normal tissue constraints, tumor progression, or other unknown reasons. Other important information was missing including pulmonary function tests, tumor location (central vs. peripheral), pack years of smoking, extent of preoperative and postoperative workup, and type of chemotherapy. However, despite the limitations of the data, there is no other study that we know that assesses the role of radiation in patients with pathologically positive nodes receiving SLR, demonstrates the incidence of pN+ in patients undergoing SLR, and shows the inferior OS of patients with pN+ undergoing SLR as compared to (bi)lobectomy.

It should be noted that the authors of this manuscript are not endorsing SLR in patients with pN+. The purpose of our investigation is to provide guidance when patients fall under these unique, less than optimal circumstances. Furthermore, because of the inferior OS associated with SLR as compared to (bi)lobectomy when pN+, we feel that aggressive surgical staging prior to surgery can spare this suboptimal outcome, especially with improving OS noted when consolidative durvalumab follows chemo/radiation in patients with N2 involvement (36).

## Conclusions

Although the incidence of pathologically positive nodes has been falling during the years of our study, positive nodes were present in close to 9% of patients undergoing SLR during the last year of our study in the small majority of patients who had one or more nodes examined. Over 40% of patients undergoing SLR had no nodes examined. Radiation, when given with adequate doses, appears to improve the OS in patients undergoing SLR with pathologic nodal positivity, especially in those with N2 nodal involvement and/or positive margins.

## Data Availability Statement

The datasets generated for this study are available on request to the corresponding author.

## Ethics Statement

Data for this study were derived from the National Cancer Database (NCDB) registry, the patient information was de-identified, thus this investigation was exempted from IRB approval.

## Author Contributions

KU, JV, IE, RV, and JF contributed to conceptualization. IE and JV contributed to data curation and contributed to the methodology and project administration. MD, KU, IE, RV, JF, DM, TF, PO, JB, RS, WW, LM, FL, and NR performed formal analysis. TF and JV contributed to funding acquisition. TF, JV, and IE contributed to procuring the resources. IE handled the software and performed validation. JV, MD, and RV supervised. All authors performed the investigation and contributed to writing the original draft. All authors contributed to writing, reviewing, and editing the final manuscript.

## Conflict of Interest

The authors declare that the research was conducted in the absence of any commercial or financial relationships that could be construed as a potential conflict of interest. The reviewer TK declared a past co-authorship with one of the author MD to the handling Editor.
