# Supplementary material for: The Incidence of Node-Positive Non-small-Cell Lung Cancer Undergoing Sublobar Resection and the Role of Radiation in Its Management
Source: Front Oncol. 2020 May 26;10:417. doi: 10.3389/fonc.2020.00417 (PMC7264374; doi:10.3389/fonc.2020.00417)

Figure 1b-d – OS in patients undergoing sub-lobar resection by node stage in patients having at least one node examined. (a) OS by node stage in patients undergoing sub-lobar resection. (b) OS by node stage by type of resection, sub-lobar vs lobectomy. Propensity match for OS by node stage by type of resection for all resected tumors(c) and only for those < 2cm in size(d). All node stages are pathologic in this figure. Figures 1b-d here include N3 nodes. Figures 1b-d in the main paper excluded N3 nodes for clarity.

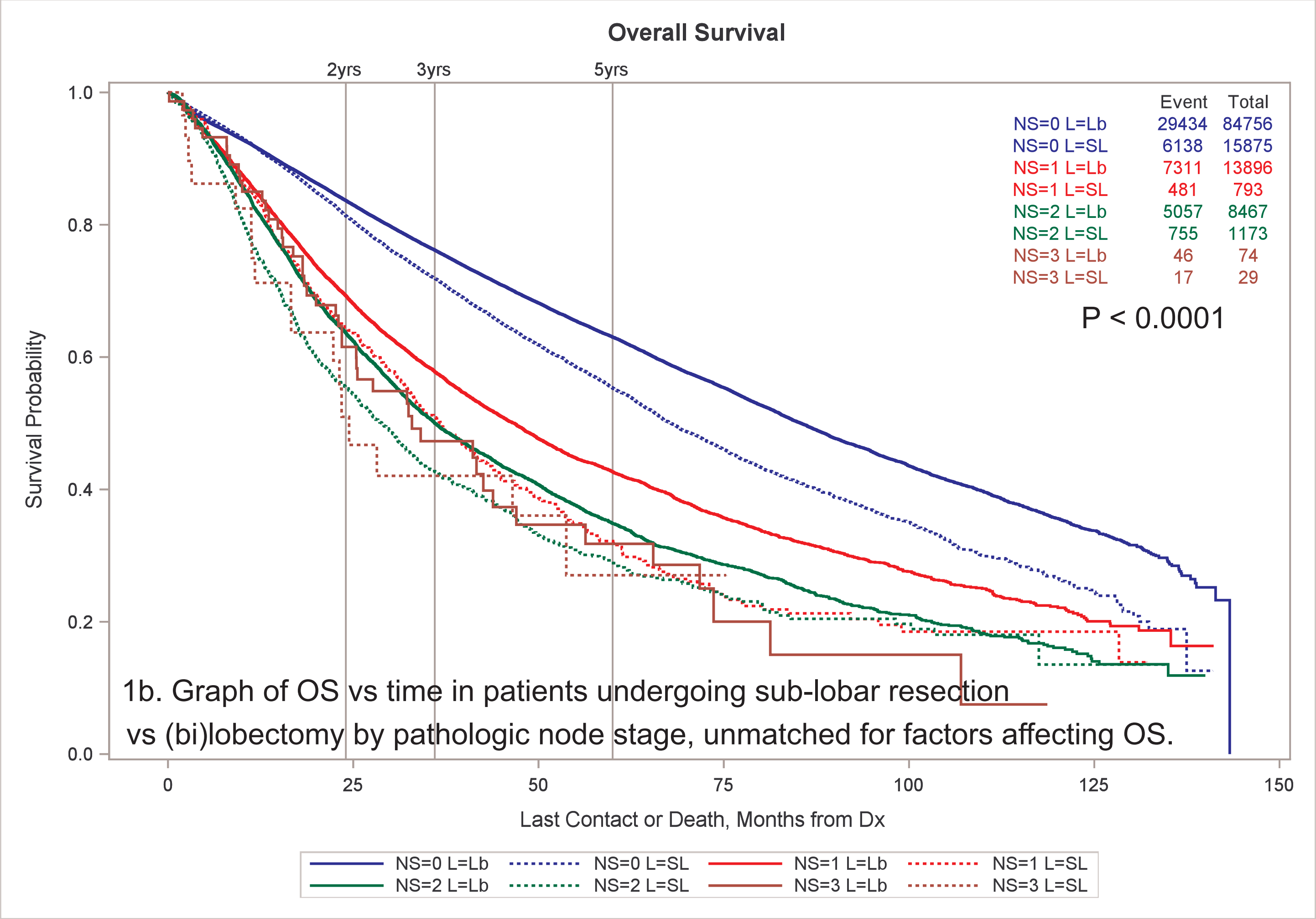

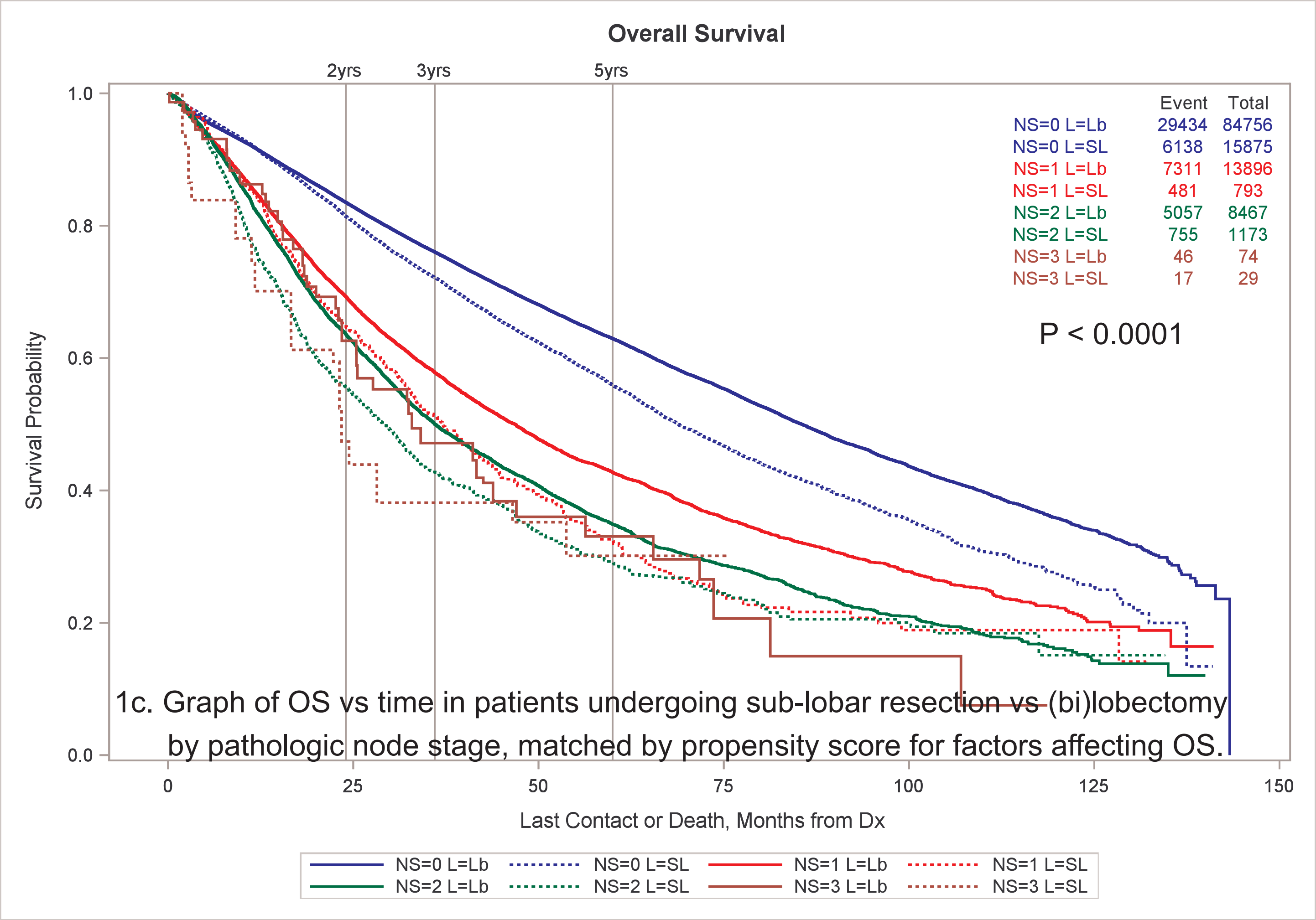

1c. Graph of OS vs time in patients undergoing sub-lobar resection vs (bi)lobectomy by pathologic node stage, matched by propensity score for factors affecting OS.

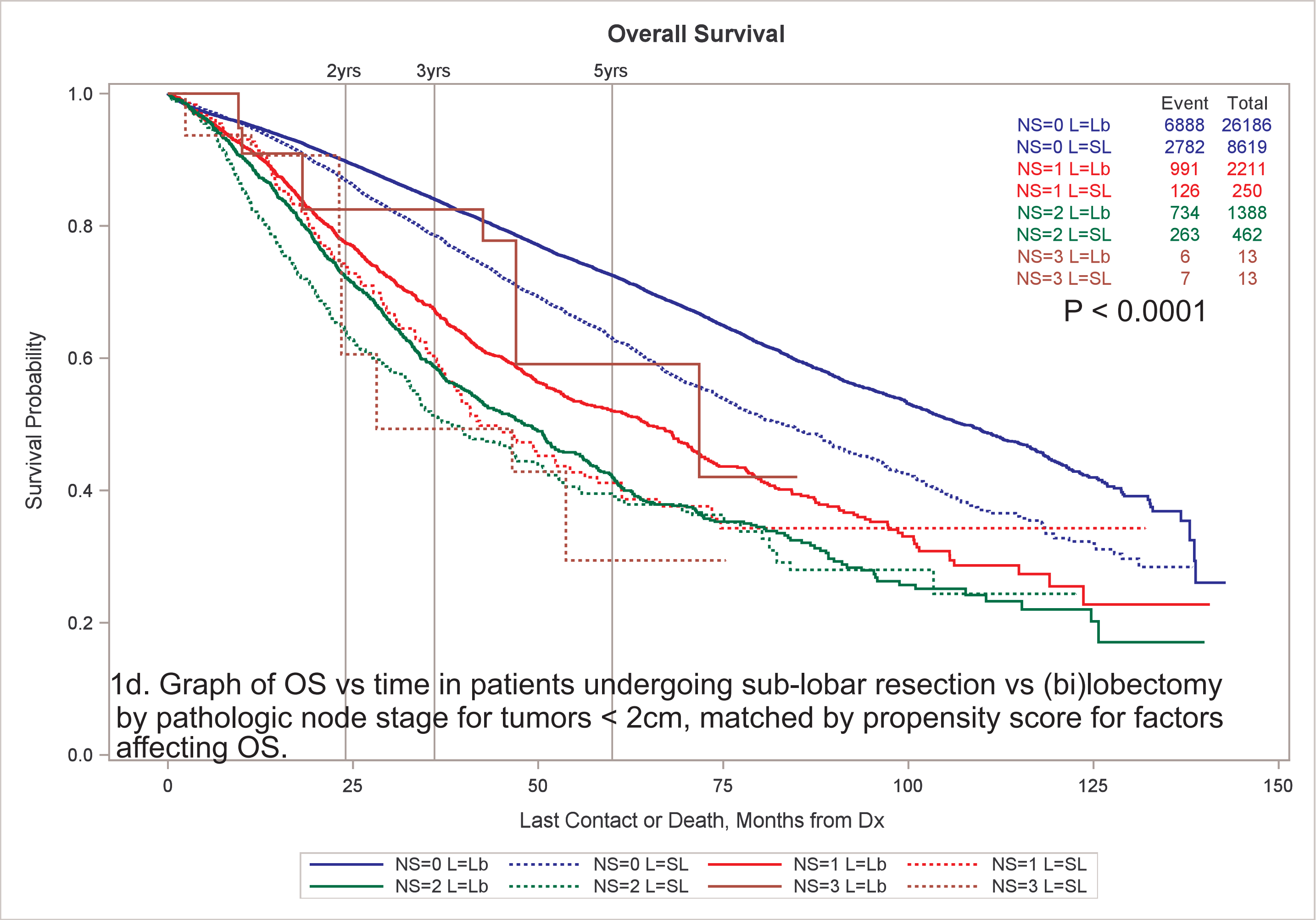

1d. Graph of OS vs time in patients undergoing sub-lobar resection vs (bi)lobectomy by pathologic node stage for tumors < 2cm, matched by propensity score for factors affecting OS.

Table 2-appendix- Percentage of patients with pathologically N1, N2, and N3 node involvement during years 2004-2014 after excluding all cases with clinically positive nodes

Year of Diagnosis	Total patients/ Year	N0 Freq (%)	Total Positive Nodes per Year (%)	N1 Freq (%)	N2 Freq (%)	N3 Freq (%)
2004	668	634 (94.9%)	34 (5.1%)	23 (3.4%)	10 (1.5%)	1 (0.1%)
2005	809	766 (94.7%)	43 (5.3%)	19 (2.3%)	23 (2.8%)	1 (0.1%)
2006	890	838 (94.2%)	52 (5.8%)	32 (3.6%)	20 (2.2%)	0 (0%)
2007	1132	1082 (95.6%)	50 (4.4%)	28 (2.5%)	22 (1.9%)	0 (0%)
2008	1902	1814 (95.4%)	88 (4.6%)	44 (2.3%)	44 (2.3%)	0 (0%)
2009	2211	2094 (94.7%)	117 (5.3%)	39 (1.8%)	78 (3.5%)	0 (0%)
2010	2452	2343 (95.6%)	109 (4.4%)	47 (1.9%)	60 (2.4%)	2 (0.1%)
2011	2651	2532 (95.5%)	119 (4.5%)	50 (1.9%)	69 (2.6%)	0 (0%)
2012	2823	2686 (95.1%)	137 (4.9%)	61 (2.2%)	75 (2.7%)	1 (<0.1%)
2013	3064	2928 (95.6%)	136 (4.4%)	49 (1.6%)	87 (2.8%)	0 (0%)
2014	3239	3108 (94.5%)	131 (4.0%)	51 (1.6%)	80 (2.5%)	0 (0%)

Table 3-appendix Multi-variate analysis for OS in the pathogenic node positive group after excluding all cases with clinically node positive nodes (n=771 remaining)

Description	Hazard Ratio	Lower 95% Wald	Upper 95% Wald	P-value
		Confidence Limit	Confidence Limit	
AGE Unit=10	1.241	1.023	1.507	0.029
SEX Male vs Female	1.537	1.234	1.914	0.0001
race: Asian or Pacific Islander vs White-Nonhispanic	0.279	0.098	0.791	0.005
race: Black vs White-Nonhispanic	0.569	0.367	0.882	
race: Other/Unknown vs White-Nonhispanic	1.006	0.685	1.478	
race: White-Hispanic vs White-Nonhispanic	0.382	0.161	0.905	
CDCC: 1 vs 0	1.148	0.911	1.447	0.21
CDCC: 2 vs 0	1.309	0.953	1.797	
Location: Left Lower Lobe vs Right Upper Lobe	1.143	0.811	1.61	0.53
Location: Left Upper Lobe vs Right Upper Lobe	1.122	0.831	1.514	
Location: Main Bronchus vs Right Upper Lobe	Undef	0	Inf	
Location: Other/NOS vs Right Upper Lobe	1.576	0.91	2.727	
Location: Right Lower Lobe vs Right Upper Lobe	1.257	0.9	1.755	
Location: Right Middle Lobe vs Right Upper Lobe	1.596	0.912	2.794	
histology: Adenosquamous vs Adenocarcinoma	1.191	0.721	1.97	0.49
histology: BAC vs Adenocarcinoma	0.825	0.47	1.448	
histology: Large Cell CA vs Adenocarcinoma	1.645	0.8	3.383	
histology: Non-small cell carcinoma vs Adenocarcinoma	1.278	0.75	2.179	
histology: Squamous Cell CA vs Adenocarcinoma	0.887	0.671	1.172	
Facility_Type: Community Cancer Program vs Academic/ Research Program	0.998	0.557	1.791	0.86
Facility_Type: Comprehensive Community Cancer Pro- gram vs Academic/Research Program	0.963	0.754	1.23	
Facility_Type: Integrated Network Cancer Program vs Academic/Research Program	0.848	0.58	1.24	

Table 3-appendix (continued) Multi-variate analysis for OS in the pathogenic node positive group after excluding all cases with clinically node positive nodes (n=771 remaining)

Description	Hazard Ratio	Lower 95% Wald	Upper 95% Wald	P-value
		Confidence Limit	Confidence Limit	
Facility_Location: East North Central vs New England	0.778	0.468	1.294	0.55
Facility_Location: East South Central vs New England	0.88	0.505	1.536	
Facility_Location: Middle Atlantic vs New England	0.65	0.397	1.065	
Facility_Location: Mountain vs New England	0.68	0.327	1.415	
Facility_Location: Pacific vs New England	0.961	0.555	1.662	
Facility_Location: South Atlantic vs New England	0.921	0.565	1.5	
Facility_Location: West North Central vs New England	0.838	0.48	1.463	
Facility_Location: West South Central vs New England	0.773	0.394	1.518	
Insurance Status: Unknown vs Private Insurance	1.333	0.586	3.034	0.33
Insurance Status: Medicaid vs Private Insurance	0.978	0.568	1.682	
Insurance Status: Medicare vs Private Insurance	0.734	0.548	0.982	
Insurance Status: Not Insured vs Private Insurance	0.748	0.222	2.521	
Insurance Status: Other Government vs Private Insurance	0.761	0.261	2.221	
Median Income 2012: $38,000-$47,999 vs <$38,000	0.831	0.59	1.169	0.065
Median Income 2012: $48,000-$62,999 vs <$38,000	0.858	0.611	1.205	
Median Income 2012: $63,000 + vs <$38,000	0.626	0.43	0.913	

Table 3-appendix (continued) Multi-variate analysis for OS in the pathogenic node positive group after excluding all cases with clinically node positive nodes (n=771 remaining)

Description	Hazard Ratio	Lower 95% Wald	Upper 95% Wald	P-value
		Confidence Limit	Confidence Limit	
Completely rural or less than 2,500 urban population, adjacent to a metro area vs Counties in metro areas of 1 million population or more	1.103	0.383	3.177	0.056
Completely rural or less than 2,500 urban population, not adjacent to a metro area vs Counties in metro areas of 1 million population or more	0.695	0.208	2.32	
Counties in metro areas of 250,000 to 1 million population vs Counties in metro areas of 1 million population or more	1.353	1.023	1.789	
Counties in metro areas of fewer than 250,000 population vs Counties in metro areas of 1 million population or more	1.255	0.852	1.848	
Urban population of 2,500 to 19,999, adjacent to a metro area vs Counties in metro areas of 1 million population or more	2.022	1.326	3.083	
Urban population of 2,500 to 19,999, not adjacent to a metro area vs Counties in metro areas of 1 million population or more	0.866	0.409	1.836	
Urban population of 20,000 or more adjacent to a metro area vs Counties in metro areas of 1 million population or more	1.033	0.616	1.734	
Urban population of 20,000 or more not adjacent to a metro area vs Counties in metro areas of 1 million population or more	1.031	0.455	2.335	
Surgery: Segmentectomy vs Wedge Resection	1.027	0.803	1.313	0.88
Surgery: Sub-Lobar Resection, NOS vs Wedge Resection	0.899	0.543	1.488	
Tumor Size (/10cm)	1.05	0.987	1.117	0.12
Any Pathologically Positive Nodes vs None	1.1	1.029	1.175	0.005
Nodes examined by pathology	0.964	0.945	0.984	0.001
Year of Diagnosis	0.995	0.934	1.061	0.88
Pathological T-Stage: T2 vs T1	1.2	0.948	1.52	0.1
Pathological T-Stage: T3 vs T1	1.198	0.757	1.897	
Pathological T-Stage: T4 vs T1	1.673	1.081	2.591	
N2 vs N1	1.24	0.981	1.567	0.072

Table 3-appendix (continued) Multi-variate analysis for OS in the pathogenic node positive group after excluding all cases with clinically node positive nodes (n=771 remaining)

Description	Hazard Ratio	Lower 95% Wald	Upper 95% Wald	P-value
		Confidence Limit	Confidence Limit	
Radiation: Any vs None	0.764	0.33	1.769	0.53
Radiation Dose (/10,000 CGY)	1.376	0.279	6.787	0.7
Chemotherapy: Any vs None	0.672	0.521	0.866	0.002
Lymphovascular invasion: Present vs Not present	1.547	1.116	2.146	0.033
lymphvasinv_3grp Unknown vs Not present	1.223	0.849	1.761	
GRADE: Cell type not determined, not stated or not applicable, unknown primaries, high grade dysplasia vs Well differentiated, differentiated, NOS	2.241	1.152	4.359	0.13
GRADE: Moderately differentiated, moderately well differentiated, intermediate differentiation vs Well differentiated, differentiated, NOS	1.42	0.936	2.155	
GRADE: Poorly differentiated vs Well differentiated, differentiated, NOS	1.595	1.039	2.447	
GRADE: Undifferentiated, anaplastic vs Well differentiated, differentiated, NOS	2.344	0.52	10.574	
Hospital readmission: Planned readmission within 30 days of discharge vs No surgical procedure of the primary site was performed, or patient not readmitted	1.272	0.721	2.245	0.072
Hospital readmission: Unknown if surgery recommended/performed, unknown if readmitted within 30 days of discharge vs No surgical procedure of the primary site was performed, or patient not readmitted	0.719	0.31	1.67	
Hospital readmission: Unplanned readmission within 30 days of discharge vs No surgical procedure of the primary site was performed, or patient not readmitted	1.725	1.109	2.683	
Surgical Discharge (/day)	1.054	1.036	1.073	<.0001

Figure 4

Patient

selection criteria.

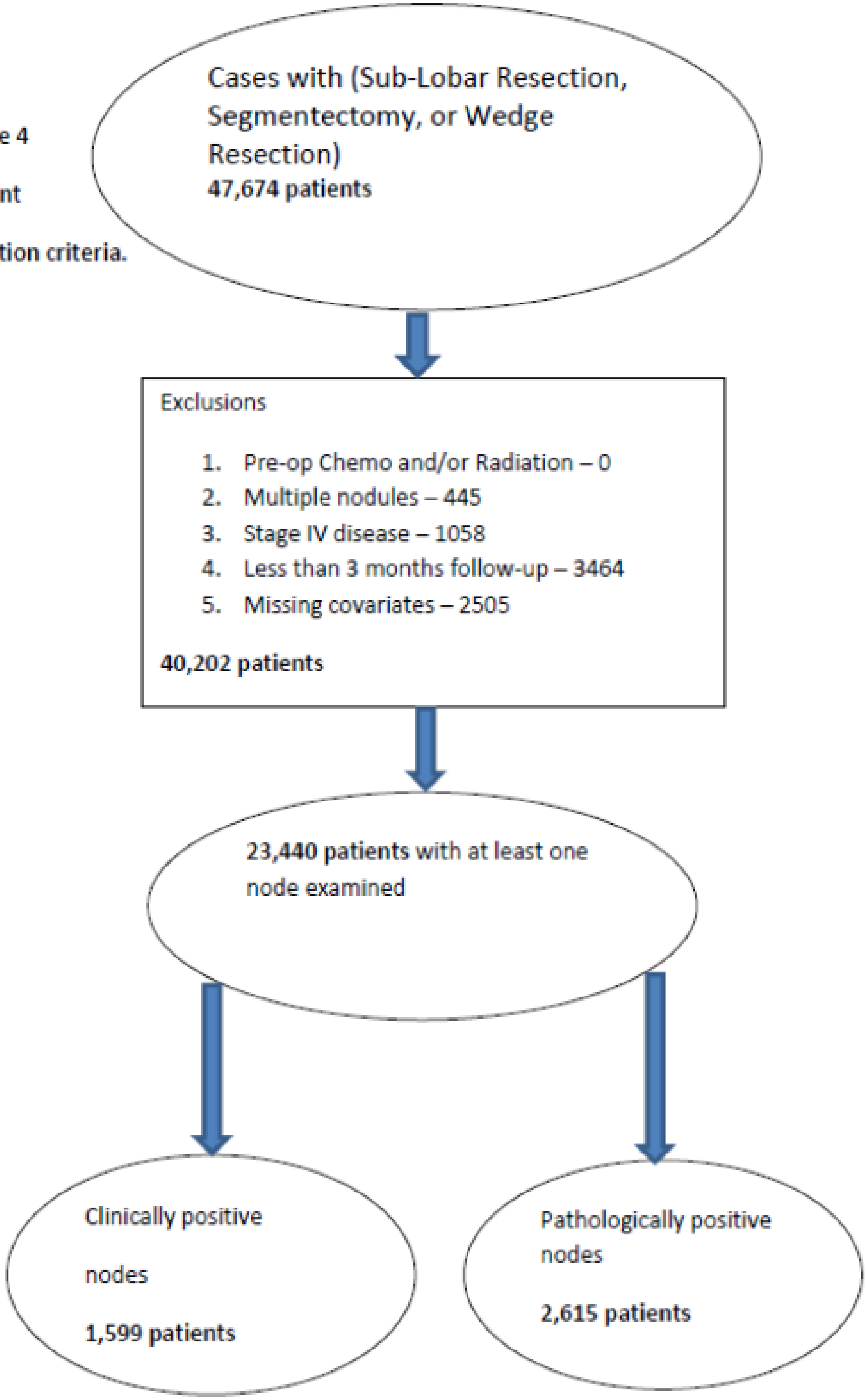

Supplement: Supplementary file 1 [file Data_Sheet_1.pdf]
